# Temozolomide, Procarbazine and Nitrosoureas in the Therapy of Malignant Gliomas: Update of Mechanisms, Drug Resistance and Therapeutic Implications

**DOI:** 10.3390/jcm12237442

**Published:** 2023-11-30

**Authors:** Bernd Kaina

**Affiliations:** Institute of Toxicology, University Medical Center, Obere Zahlbacher Str. 67, D-55131 Mainz, Germany; kaina@uni-mainz.de

**Keywords:** glioblastoma, temozolomide, procarbazine, CCNU, therapy, drug resistance

## Abstract

The genotoxic methylating agents temozolomide (TMZ) and procarbazine and the chloroethylating nitrosourea lomustine (CCNU) are part of the standard repertoire in the therapy of malignant gliomas (CNS WHO grade 3 and 4). This review describes the mechanisms of their cytotoxicity and cytostatic activity through apoptosis, necroptosis, drug-induced senescence, and autophagy, interaction of critical damage with radiation-induced lesions, mechanisms of glioblastoma resistance to alkylating agents, including the alkyltransferase MGMT, mismatch repair, DNA double-strand break repair and DNA damage responses, as well as IDH-1 and PARP-1. Cyclin-dependent kinase inhibitors such as regorafenib, synthetic lethality using PARP inhibitors, and alternative therapies including tumor-treating fields (TTF) and CUSP9v3 are discussed in the context of alkylating drug therapy and overcoming glioblastoma chemoresistance. Recent studies have revealed that senescence is the main trait induced by TMZ in glioblastoma cells, exhibiting hereupon the senescence-associated secretory phenotype (SASP). Strategies to eradicate therapy-induced senescence by means of senolytics as well as attenuating SASP by senomorphics are receiving increasing attention, with therapeutic implications to be discussed.

## 1. Introduction

Malignant brain tumors (high-grade glioma, WHO grade 3 and 4) have a dismal prognosis. They grow infiltratively, and recurrences are common [1]. Glioblastoma (GBM), the most frequent and aggressive brain cancer, has had rising incidence rates in recent years [2,3]. The tumor remains incurable, with a median survival time of 15 months and a 5-year survival of 7.2% [4]. The standard of care rests on surgical resection, followed by radiochemotherapy and adjuvant chemotherapy. The GBM gold standard treatment has remained unchanged since 2005 [5], despite the therapeutic outcome being unfavorable. Different non-approved protocols are used [6] in the first and second recurrent situations. 

In this setting, the first-line therapeutics are still methylating and chloroethylating agents. Historically, the situation has been a paradox, as methylating nitrosoureas are powerful neurotropic carcinogens. Thus, the highly potent alkylating agent N-methyl-N-nitrosourea (NMU) produces brain tumors with high frequency directly and also transplacental in rats [7] and was used for chemotherapy in the early 1970s, albeit without much success [8,9]. This nitrosourea was replaced by other DNA-methylating substances that are less aggressive and therefore easier to handle pharmacologically. These include procarbazine (Natulan), dacabazine (DTIC), streptozotocin (STZ, Zanosar), and the last-generation drug, temozolomide (TMZ, Temodar) [10]. Today, the methylating triazene derivative TMZ is recommended as first-line therapy in IDH wild-type glioblastoma by the European Association of Neuro-Oncology (EANO) and the Society of Neuro-Oncology [11,12]. Procarbazine, which is administered together with lomustine (1-(2-chloroethyl)-3-cyclohexyl-1-nitrosourea: CCNU, Gleostine) and vincristine (Oncovin) in the PCV scheme, is considered to be first-line therapy in IDH-mutant glioblastoma [11]. Procarbazine is a prodrug that needs to be activated metabolically, and this occurs predominantly in the liver by cytochrome P450 (involving CYP3A4), as TMZ spontaneously decomposes in aqueous solutions into the reactive metabolite. In both cases, nucleophilic diazonium ions are generated, which methylate RNA and DNA at all nucleophilic sites. In the DNA, 12 alkylation products, including pyrimidines and purines, were described [13]. TMZ is administered in postoperative primary therapy together with radiation, as well as in subsequent maintenance therapy [14]. TMZ therapy can be extended with daily administration over long periods of time since the drug is relatively well tolerated [15]. 

CCNU (N-chloroethyl-N-cyclohexyl-nitrosourea; lomustine) is a representative of the chloroethylating nitrosoureas (including also carmustine and nimustine). It is primarily used in the recurrent situation of glioblastoma as well as in the therapy of low-grade gliomas together with procarbazine (PCV scheme) [16]. CCNU breaks down spontaneously in the cell, with the reactive metabolites adding chloroethyl groups to the DNA at various sites. One of the DNA alkylation products of methylating and chloroethylating drugs is O^6^-alkylguanine, and therefore, this group of genotoxins, comprising widely distributed environmental carcinogens such as N-nitrosodimethylamine, is referred to as O^6^-alkylating agents (O6AA) [17]. The activation of procarbazine by cytochrome P450 in the liver and the slow spontaneous decomposition of TMZ result in a relatively slow influx of reactive metabolites (compared to the highly reactive NMU), causing the accumulation of DNA adducts in repair-defective tumor cells, while they are repaired in normal, repair-competent tissue. 

In order to find out why glioma chemotherapy is quite ineffective, it is necessary to understand how the therapeutic agents work at the cellular and molecular level. This review aims to discuss the mechanisms of methylating and chloroethylating agents, focusing on MGMT and other DNA repair processes, DNA damage response (DDR), apoptotic pathways, induction of autophagy, and senescence. Additionally, the question to ponder is why gliomas are difficult to cure with these drugs, including mechanisms of drug resistance and apoptosis-preventing pathways, pointing to the need for repair inhibitors, senolytics, senomorphics, and other strategies to overcome chemoresistance.

## 2. Key Node MGMT

Accumulation of the pre-toxic damage to O^6^-alkylguanine occurs when the repair protein O^6^-methylguanine-DNA methyltransferase (MGMT) is not expressed. This applies to 20–40% of all glioblastomas [18]. MGMT is otherwise expressed in all tissues, although at variable levels. High expression is found in the liver, intestines, and also in peripheral lymphocytes, and only low levels are found in CD34+ hematopoietic stem cells [19]. This explains the hematotoxic side effects, which are sometimes therapy-limiting. Rats lack MGMT in brain tissue, which explains the neurotropic carcinogenesis triggered by methyl- and ethylnitrosourea [20]. In humans, there is debate as to whether the low MGMT levels in brain tissue could be the cause of the development of brain tumors [21].

The different expression of MGMT in the tissues as well as the strong inter-individual variability [22] indicate that this repair protein is subject to strong regulation [13,19]. This occurs at the transcriptional and post-transcriptional levels through phosphorylation and poly-(ADP-ribosyl)ation (PARylation) as well as through interaction with other proteins, including poly (ADP-ribose) polymerase-1 (PARP1), the transcription factor SP1, and p53. At the gene level, regulation occurs transcriptionally by SP1, AP1, and the glucocorticoid receptor, among others, and epigenetically by CpG methylation in 5-methylcytosine islands in the promoter region of the gene [23]. Hardly any other repair genes are as strongly epigenetically deregulated as MGMT [18]. This seems to be the case in particular in glioblastomas, since about 20% of tumors lack MGMT enzyme activity and about 40% are methylated in the promoter, as shown by methylation-specific PCR assays [24]. Of note, most GBM have lost one copy of chromosome 10 that harbors the MGMT gene; hence, promoter methylation of the remaining copy may completely abolish MGMT expression. An interesting question that arises in this context is whether epigenetic downregulation of MGMT already occurs in glioblastoma progenitor cells, which would be causally related to malignant transformation since O^6^-methylguanine is a highly potent premutagenic and precarcinogenic lesion [25]. Although numerous studies have revealed MGMT as a prognostic marker of therapy response [26] and therapy stratification in elderly patients with glioblastoma rests on MGMT [27], it should be noted that there are also studies indicating inconsistencies associated with patients’ outcomes [28], indicating the need for highly standardized methylation-specific PCR in order to avoid biased results. Alternative methods such as high-resolution melt (HRM) analysis have been described and recommended for routine screenings [29] that might be included in comparative clinical studies.

## 3. Mechanism of Cytotoxicity of O6AA: Upstream Pathways

**Figure 1:**
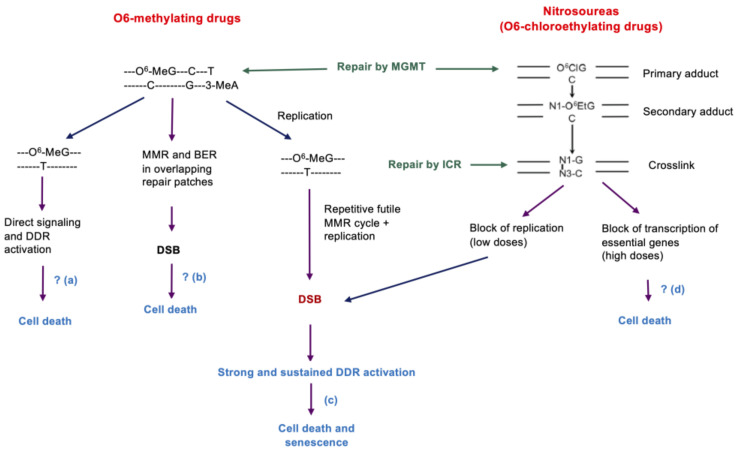
Comparison of methylating and chloroethylating anticancer drugs. (**a**) This model is based on the finding that MMR proteins (MSH2/MSH6) recognize O^6^MeG/T, resulting in recruitment of ATR-ATRIP to the mismatch, thus activating the ATR kinase and CHK1. This was shown in cell extracts [33], but evidence is lacking that the signal is strong enough to activate apoptotic pathways, which would occur in the treatment cell cycle. (**b**) This model rests on the observation that MMR proteins can recognize to some extent O^6^MeG/C and, together with BER activity, DSBs can be formed, which was shown on plasmid DNA in vitro [34]. Evidence is lacking that this applies to in vivo. The model is incompatible with the replication dependence of TMZ-induced cell death. (**c**) The futile MMR cycle model [35] claims that damage tolerance requires MMR, which is associated with replication arrest [36]. This implicates that DSB are formed and cell death through apoptosis occurs in a replication-dependent manner in the second post-treatment S-phase, which was experimentally confirmed [37,38]. (**d**) Although it was shown that block of transcription activates the DDR [39], it is unlikely that therapeutic doses trigger cell death through this mechanism.

A large number of studies on different cell systems and animal models have shown that cells that do not express MGMT are highly vulnerable to O^6^-methylating compounds [13,30,31]. The sensitivity was shown to be inversely proportional to the amount of MGMT protein (or number of MGMT molecules) per cell [32]. This also applies to chloroethylnitrosoureas. The O^6^-chloroethylguanine adduct, like O^6^-methylguanine, is repaired by MGMT through direct alkyl group transfer to a cysteine residue in the active site of the MGMT molecule. However, the mechanisms of cytotoxicity for unrepaired O^6^-methylguanine and O^6^-chloroethylguanine are fundamentally different. In the first case, during replication, an O^6^MeG/thymine mismatch, which is a substrate for a malfunctioning mismatch repair cycle, is formed. This creates gaps in the DNA, which lead to DNA double-strand breaks (DSBs) in the subsequent replication cycle (Figure 1). These DSBs are the ultimate triggers for initiating survival and cell death programs. Unlike O^6^MeG, the O^6^-chloroethylguanine lesion is not stable. It undergoes an intramolecular transformation several hours after induction, ultimately forming a DNA interstrand crosslink. These crosslinks block DNA replication as well as transcription (Figure 1). In PCV therapy, a crosstalk between both pathways may lead to synergistic effects, which are ameliorated by vincristine, which causes mitotic death in the surviving, proliferating fraction.

**Figure 2:**
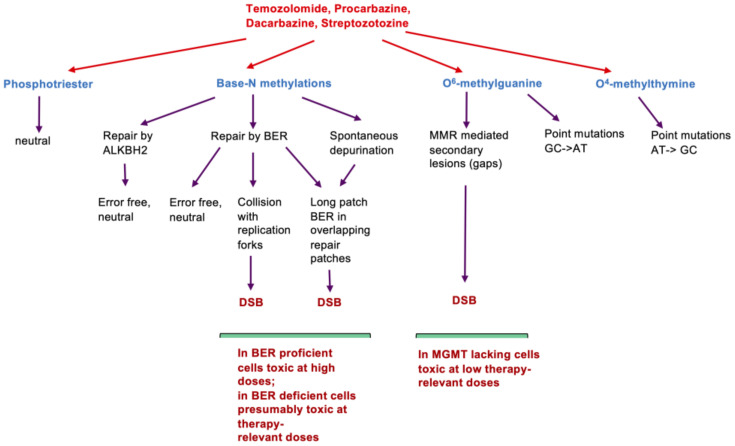
DNA damages and their consequences following treatment with O^6^-methylating agents.

In the low-dose range of CCNU, which is therapeutically relevant, the inhibition of replication and the associated formation of DSB are very likely the crucial events, while the inhibition of transcription becomes important at high dose levels [40]. For methylating drugs and very likely also for chloroethylating nitrosoureas, DNA replication is essential for DSBs and the induction of cell death (for DNA lesions induced by methylating drugs and their processing, see Figure 2). Since O^6^-alkylguanine is the only upstream trigger, MGMT is the key node in defense through rapid and error-free repair of the critical primary lesion. 

## 4. Downstream Events: Signaling Pathways and Apoptosis

The signaling pathways that are triggered by O^6^MeG in normal and tumor cells are well described [41]. It was shown on synchronized cells that TMZ induces DSBs in the second cell cycle after treatment (i.e., following two DNA replication cycles) [37,42]. These lesions, as well as the inhibition of DNA replication at O^6^MeG/T-MMR clusters, activate the PI-3 kinases ATR and ATM, which in turn activate CHK1 and CHK2 by phosphorylation. In this process, the ATR kinase seems to play a dominant role [43]. One target of the activated kinases is the p53 protein, which, as a tetrameric transcription factor, regulates genes that control apoptosis pathways [44]. It also regulates genes whose products are involved in DNA repair [23]. The balance in the expression of these genes is crucial in determining the cell’s fate, life or death. 

**Figure 3:**
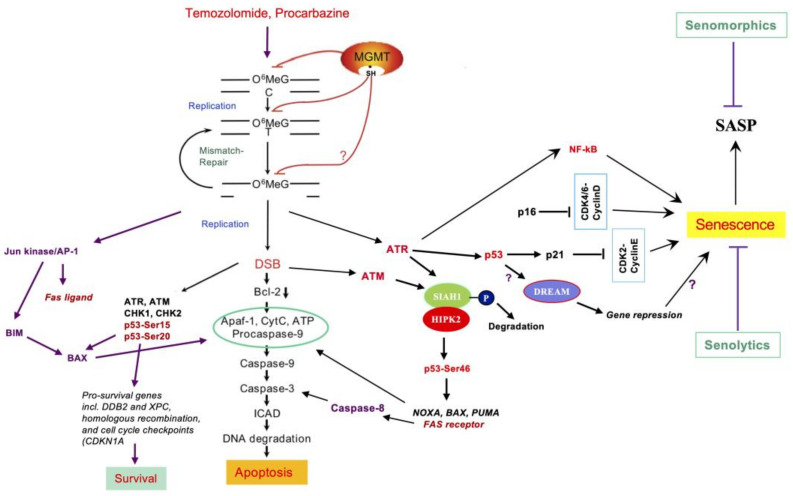
Mechanism of cell death and senescence triggered by the critical lesion O^6^-methylguanine. A hallmark is p53-independent Bcl-2 decline [42], stimulating mitochondrial apoptosis. p53 regulates the HIPK2-p53Ser46 pathway, activating Fas-dependent apoptosis [45]. O^6^MeG-derived lesions also activate AP-1, which targets Fas-L [47] and stimulates the BIM-BAX apoptosis pathway [46]. Blocked replication forks and DSBs in the 2nd replication cycle after treatment give rise to a sustained ATR and ATM activation, which activates both survival [43] and death [45] pathways. It is important to note that a robust target of p53Ser15,20 is achieved by the repair genes encoding DDB2 and XPC [48], which, however, do not repair DNA methylation lesions, but are essential for the repair of crosslinks. O^6^MeG triggers also the ATR/ATM-HIPK2-p53Ser46 pathway [45] and DDR-dependent senescence [49]. Senolytics causing senescent glioma cell clearance [50] and senomorphics attenuating the SASP [51] are considered supportive therapy with O6AA. For further explanations, see text.

In cancer therapy, it is desired that cell death pathways dominate. Here, p53 plays a key role as a “decision maker” too, because p53 is phosphorylated at various sites, which influences its effect as a transcription factor. Phosphorylation at serine 15 of the protein preferentially activates repair genes, such as *DDB2* and *XPC*, while phosphorylation at serine 46 stimulates pro-apoptotic genes, such as *FAS* and *BAX* (Figure 3). Importantly, another kinase involved in the formation of p53Ser46 is HIPK2. This enzyme is inactive due to binding to the inhibitor SIAH1. The activation of ATM and ATR through O^6^MeG-generated secondary lesions leads to the phosphorylation of SIAH1, which releases HIPK2 and thus phosphorylates p53 (Figure 3). We showed in glioblastoma cells that therapeutically relevant doses (<50 µM) of TMZ are effective in activating the ATR/ATM-SIAH1/HIPK2-p53Ser46 axis and thus trigger apoptosis [45]. In addition to this signaling pathway, the JUN kinase pathway is also activated, which regulates both receptor-mediated and mitochondrial apoptosis via the FAS ligand (FAS-L) and BIM [46]. The signaling pathways triggered by O^6^MeG giving rise to apoptosis and cellular senescence (described below), are outlined in Figure 3.

There is some work on necrosis induced by TMZ, notably in the context of ROS [52]. Programed necrosis (necroptosis) is bound to ATP depletion, which occurs following extensive BER and PARP-1 activation. Although TMZ and procarbazine induce N-methylations that trigger BER, the amount at therapeutic dose levels seems to be insufficient to induce necroptosis. However, in drug-resistant MGMT-proficient cells, for which higher doses are required to elicit cytotoxic effects, necroptosis may come into play. The clinical significance and therapeutic potential of necroptosis, which may cause inflammation following O6AA+RT (presumably visible as pseudoprogression), remain uncertain [53].

## 5. TMZ-Induced Cellular Senescence

Initially, focusing on O^6^MeG-triggered cell death through apoptosis, we believed this to be the therapeutically most important event following treatment with O6AA [54]. We later found that O^6^MeG induces not only apoptosis, but also cellular senescence (CSEN) [49]. In glioblastoma cells in vitro, apoptosis and senescence are late events that become detectable not earlier than 4 days after TMZ treatment in proliferating cultures [55]. In these experiments, senescence runs parallel to apoptosis, but with a significantly higher yield (20% apoptosis and 60% senescence, as measured by Annexin V/PI and SA-ßGAL flow cytometry, respectively). Senescence peaks 6–10 days after TMZ exposure. Under optimal conditions, about 90% of the cells in the population are in the senescence state, which is evident by SA-ßGAL staining and morphological features (giant cells, large nuclei, polyploidy), while apoptotic cells have been eliminated. The remaining senescent cells are arrested in the G2 phase, showing high levels of intracellular ROS, oxidative DNA damage, and DSBs (γH2AX/53BP1 foci) that are not preferentially located in telomeres [55]. Although the DSB level is high, senescent cells can repair DSBs, indicating that these are “stabilized” DSBs formed during senescence induction. In this scenario, O^6^MeG is the primary trigger, but the damage is not required for maintaining the senescent status, as revealed by our work with inducible MGMT [55]. TMZ-induced senescence was initiated by damage recognition through the MRN complex, activation of the ATR-CHK-1 axis, and degradation of CDC25c, causing G2/M arrest. It required functional p53 and was dependent on sustained p53(ser15)–p21 induction. p14 and p16, for which glioblastoma cells are frequently mutated, were not required [56]. TMZ-induced senescent glioblastoma cells are characterized by NF-kB upregulation and high-level production of inflammatory cytokines such as IL-6 and IL-8 [56], which is a hallmark of the senescence-associated secretory phenotype (SASP) [57]. The pro-inflammatory property of senescent cells is considered to be the driving force in tumorigenesis and tumor progression [58]. TIS frequently goes along with the repression of a battery of genes that are obviously not needed for maintaining viability status. Thus, several repair genes not required under non-proliferating conditions were shown to be downregulated in TMZ-induced CSEN [56]. The DREAM complex is a master regulator of gene repression in senescent cells [59]. Its involvement in CSEN in gliomas has not been shown yet (Figure 3).

There is little work showing that apoptosis and senescence occur in vivo, in the tumor tissue, after TMZ. We tried to approach the question by determining apoptosis (TUNEL assay) and senescence (γH2AX and H3K27me3 positive cells) in the primary tumor and in the corresponding recurrence. The proportion of senescent cells was significantly higher in the recurrences for both markers, while the apoptosis rate was lower [55]. Since TMZ-induced senescent cells are supposed to not be eliminated during the therapy, the result matches the expectation: a large proportion of the glioblastoma cells remain in the senescent stage after RT/TMZ and are still present in the recurrence.

## 6. Autophagy

O^6^MeG is also an autophagy-inducing lesion, with the upstream pathways needing MMR and a lack of MGMT, thus being identical to the end-points of apoptosis and senescence [49]. The inhibition of autophagy increased the rate of TMZ-induced apoptosis. Autophagy is therefore considered a protective function in glioblastoma cells treated with TMZ, at least in the therapeutic dose range [49]. An effective inhibitor of autophagy is chloroquine. In cultivated glioma cells, chloroquine ameliorated the yield of apoptosis following TMZ treatment [60,61], and various efforts have been made to repurpose the anti-malaria and anti-inflammatory drug for cancer therapy including GBM [62]. Chloroquine, which is able to cross the blood–brain barrier, was tested in several clinical trials and was found to extend patient survival after radiochemotherapy that included the O^6^-alkylator/crosslinker carmustine (BCNU, Gliadel) [63] and RT alone in the recurrent state [64]. A study based on a larger group of patients and TMZ standard therapy (RT+TMZ) revealed strong dose-limiting toxicity without improved overall survival [65]. A phase I trial determined the maximum tolerated dose of chloroquine in the chemoradiation setting and encouraged further clinical trials with O6AA [66]. Other inhibitors of autophagy are in preclinical investigation, which ameliorate glioblastoma cell death when combined with chloroquine and TMZ [67] or have additionally alkylating properties [68].

## 7. Are There TMZ Thresholds?

A criticism sometimes put forward to explain the low therapeutic potency of TMZ is to argue that, in some experimental work, TMZ concentrations of 1000 up to 4000 µM are used, whereas much lower concentrations are measured in the serum (maximally up to 50 µM) and even less in the tumor (1.5 to 35 µM), which explains the argument that in vitro data cannot be translated to the in vivo situation [69]. In fact, there are a number of papers that mention the use of TMZ in the high-dose range referred to above. We measured cell death and signaling over a long period after adding TMZ to the medium (which has a short half-life of about 2 h). At the early stage (up to 3 days after treatment), O^6^MeG-triggered events are not yet visible, as this requires longer exposure times. Therefore, the early effects observed by authors using high TMZ doses (>200 µM) are likely due to base N-alkylations. As these are removed through base excision repair (BER), high doses are needed to create enough lesions to activate the damage response. In contrast, the O^6^MeG response detectable after >72 h is already emerging in MGMT-deficient cells at doses <50 µM [55]. 

Dose–response curves also showed that apoptosis and senescence in gliobastoma cells increase linearly and display no threshold. This also applies to p53 (ser 15 and Ser46) activation [70,71]. This unexpected finding is noteworthy because it deviates from the paradigm that low doses activate p53 protective functions and high doses induce cell death. Apparently, survival functions, cell cycle arrest, senescence, and apoptosis are all activated in the glioblastoma population in an overlapping manner. How the decision is made in the individual cells between survival, senescence, and death triggered by the same DNA damage (see Figure 3) is still a mystery.

## 8. Damage Accumulation in MGMT-Lacking Cells

In tumor therapy, TMZ is administered daily. In MGMT-lacking tumor cells (promoter-methylated), the critical adduct O^6^MeG is expected to accumulate following repeated treatments, as it is not repaired. Does an accumulation of the toxic effect also occur under these circumstances? We investigated this question in dose-fractionated experiments by treating glioblastoma cells daily with 5 µM TMZ (which corresponds to the intratumoral concentration) for 5 days and compared this with a single dose of 25 µM cumulatively (this corresponds approximately to the serum level). We observed an accumulation of DSB, apoptosis, and senescence, with cumulative doses approaching the effect of a single high dose [71]. Although we are not aware of data on the amount of O^6^MeG in the tumor tissue after TMZ treatment, it is reasonable to assume that O^6^MeG also accumulates in the MGMT-lacking tumor, leading to additive effects even when a low-dose (metronomic) schedule is used. 

## 9. How Much O^6^MeG Is Required for Inducing DSBs, Cell Death, and Senescence?

It was shown that in A172 glioblastoma cells, a dose of 20 µM TMZ induces 14,000 O^6^MeG adducts per cell, which produced 32 DSB (measured as γ-H2AX and 53BP1 colocalized foci), 12% cell death, and 35% senescence in the population. In LN229 cells, the same dose induced 20,600 O^6^MeG adducts, 66 DSBs, 24% cell death, and 52% senescence [71]. Since the dose roughly corresponds to the achievable target concentration, it is concluded that in the therapeutic situation, significant amounts of O^6^MeG are induced in the tumor, which cause effects. The prerequisites are MGMT deficiency, cell proliferation, MMR competence, low DSB repair, activation of DDR, and apoptosis cascades.

## 10. Can TMZ Cause Toxicity in MGMT+ (Promoter-Unmethylated) Cells? 

MGMT is inactivated by the repair reaction and takes some time (days) to regenerate. With the daily administration of TMZ, it is conceivable that MGMT becomes depleted and O^6^MeG accumulates in unmethylated tumors. However, TMZ-mediated MGMT inactivation is likely therapeutically less efficient, as the toxic lesion O^6^MeG is used up unnecessarily for MGMT depletion, at least in the first treatment cycles. Actually, the treatment of newly diagnosed GBM with a dose-dense TMZ scheme that depletes MGMT in lymphocytes did not improve the therapeutic outcome, regardless of the methylation status [72].

TMZ-mediated MGMT depletion might be involved even in promoter-methylated cases since a complete lack of MGMT in methylated tumors is an unproven assumption. CpG methylation in the MGMT promoter occurs in different islets, and it is not clear if a positive PCR reaction in a selected promoter segment is indicative of MGMT being completely silenced. In a study on glioblastomas, in which we determined MGMT activity and promoter methylation in the same specimens, about 20% of the samples were completely lacking MGMT, whereas 40% were methylation positive [73]. The CpG methylation pattern was shown to be characteristic for each GBM and associated with very low transcripts and undetectable MGMT activity [74]. Since even small amounts of MGMT can be protective, metronomic and long-lasting administration is presumably beneficial both in promoter-methylated and unmethylated cases [75]. 

## 11. TMZ-Mediated Upregulation of MGMT?

An important question is whether MGMT is upregulated after DNA damage by TMZ and CCNU (as well as after irradiation). In rat liver and hepatocytes, MGMT can clearly be induced by genotoxic treatments [76]. In human glioblastoma cells, however, we were unable to detect any induction at doses in the therapeutic range, but we observed the upregulation of MGMT by glucocorticoids, which is in line with the presence of GREs in the MGMT promoter [77]. The finding is of particular interest since corticosteroids like dexamethasone are frequently used during O6AA therapy, and clinical studies show a negative correlation between use and therapy success [78]. In GBM spheroids, some induction of MGMT was evident, albeit at higher dose levels than used in the studies cited above [79]. The epigenetic regulation of MGMT has been extensively reviewed before [18]. 

## 12. MGMT Inhibition and Chemoprotection

It should be noted that all tissues in the human body, except presumably the brain of some individuals [21], harbor MGMT. Low activities are found in CD34+ blood stem cells [19]. These are particularly sensitive, especially since the MGMT resynthesis, which requires 1–2 days in lymphocytes, does not compensate for the depletion following daily TMZ administration. The hematotoxic therapy-limiting side effects are very likely to be attributed to this. Hematotoxicity due to low CD34+ cell protection may explain one of the limitations of MGMT inhibitors. The pseudosubstrates O^6^-benzylguanine (O6BG) and O^6^-bromothenylguanine (lomeguatrib) are extremely efficient in MGMT inactivation and have no side effects [80]. However, together with TMZ or nitrosoureas, the systemic side effects are so strong that they require a dose reduction, which in turn limits the therapeutic effect. Hematopoietic stem cell protection would be a reasonable strategy. This can be achieved by the transfer of MGMT cDNA that encodes MGMT protein and is still able to repair O^6^MeG, but is resistant to the pseudosubstrates. Although preclinical studies demonstrated feasibility and clear stem cell protection after treatment with different O6AA [81], the approach did not reach clinical trials. Tumor targeting (we approached this via covalent binding of O^6^BG to glucose [82,83,84]) or local administration into the tumor cavity are alternative strategies. Intracerebral administration via an Ommaya reservoir turned out to be feasible and free of side effects [85]. Since cytotoxicity mediated by O^6^MeG is proliferation-dependent, the toxic effects brought about by intracerebral high-dose administration are expected to be restricted to the proliferating glioma tissue.

An alternative strategy of tumor sensitization through MGMT inhibition is based on targeting bromodomain and extra-terminal tail (BET) proteins, which have been identified as potential epigenetic targets in cancer, including glioblastoma. The inhibition of these epigenetic modifiers was shown to reduce MGMT without affecting MMR protein expression [79]. Actually, an inhibitor of BET (Trotabresib) was shown to have antitumor activity in patients with high-grade gliomas and was well tolerated in the TMZ+RT concomitant and TMZ adjuvant settings [86]. Further clinical trials are warranted to establish this new approach in GBM therapy, notably for promoter-unmethylated cases.

## 13. IDH1

For diffuse astrocytic glioma, the 2021 5th edition of the WHO Classification of Tumors of the Central Nervous System reflects the discovery of molecular markers underlying neoplasms of the central nervous system, such as in isocitrate dehydrogenase 1/2 (IDH1/IDH2), ATP-dependent helicase ATRX (ATRX), histone H3-K27M, and the 1p/19q chromosomal deletion [87]. IDH mutations were found in about 70% and 5% of grade III and grade IV gliomas, respectively [88]. Since IDH significantly correlates with the prognosis, IDH-wt gliomas are now classified as CNS WHO grade 4 [88]. IDH mutant gliomas are considered a separate tumor entity that is genetically and epigenetically different from GBM.

The enzymes IDH1 and IDH2 catalyze the oxidative decarboxylation of isocitrate to alpha ketroglutarate (α-KG), also known as 2-oxoglutarate. Although mutations of either *IDH1* or *IDH2* in gliomas are heterozygous, they have an impact on drug resistance as both IDH1 and IDH2 form homodimers to exert their catalytic function and mutant homodimers and heterodimers are inactive [89]. Nearly all the identified mutations have been a single amino acid missense mutation in *IDH1* at arginine 132 (R132) or the analogous residue in *IDH2* (R172) [90]. IDH1/2 mutations result in the abnormal production of 2-hydroxyglutarate (2-HG) instead of α-KG. 2-HG was found to inhibit the enzymatic function of many α-KG-dependent dioxygenases, including histone demethylases and DNA demethylases (Ten-eleven translocation family), causing widespread changes in histone and DNA methylation and potentially promoting tumorigenesis [91,92]. Importantly, patients suffering from an IDH-mutated glioma have prolonged progression-free survival (50 months PFS) and overall survival (OS) compared to IDH wild-type glioma (7.8 months PFS) patients [90,93,94]. 

Since the discovery of the *IDH1/2* mutations, efforts have been undertaken to explore them for an improved treatment. Thus, it was believed that IDH-mutated tumors are more sensitive to chemotherapy by TMZ [95,96] or RT [97,98]. However, mutant IDH1 was shown to lead to TMZ resistance by upregulating homologous recombination [99], while others found that IDH mutations suppress homologous recombination [100]. More strikingly, there was no difference between IDH mutations and wild-type tumors as to TMZ sensitivity (ref [98,101] and our own unpublished data). However, radiation decreased the viability of IDH-mutated cells more than their wild-type counterparts, and ROS levels were always higher in IDH-mutated cells after chemo and/or radiation treatment, even in untreated cells [98,102,103,104,105]. Increased ROS levels are believed to be the cause for the observed phenotypes of these cells. These circumstances are reflected in the guidelines for the treatment of gliomas (endorsed by the SNO) recommending treatment of primary IDH-mt tumors with PCV, and IDH-wt glioblastomas with TMZ [88]. Thus, IDH-mt gliomas may take advantage of impaired repair of crosslinks induced by CCNU (see Figure 1) and mitotic catastrophe resulting from vincristine.

## 14. EGFR Mutation/Amplification

GBMs often have elevated levels of receptor tyrosine kinases, which result from EGFR amplification or mutation (EGFRvIII) [106,107,108]. Also, PDGFR, FGFR, and aberrant PI3K/AKT signaling can be involved [107]. EGFRvIII seems not to be associated with PFS and OS after TMZ-based therapy, while it was associated with a better therapeutic response to CDK inhibitors [109]. Thus, it seems that EGFR mutation/amplification does not significantly contribute to sensitivity to O^6^-alkylating drugs.

## 15. Dual Role of p53

According to the cancer genome map (TCGA), p53 is mutated in 31% of glioblastomas and 48% of low-grade gliomas. The p53 protein has an impact on drug sensitivity through (a) transcriptional activation of survival/repair genes, (b) transcriptional activation of apoptosis and senescence genes, and (c) regulation of apoptosis through protein interactions. 

(a) As outlined above, TMZ and CCNU are potent activators of p53, which occurs via the kinases ATR-CHK1 and ATM-CHK2 as well as HIPK2. This results in the phosphorylation of p53Ser15, p53Ser20, and p53Ser 46, among others [45]. p53Ser15 and Ser20 cause the upregulation of p21 and thus G1/S cell cycle blockade. According to the classical paradigm, this allows more time for pre-replicative repair, thereby reducing the toxic effect. However, in MGMT-deficient cells, in which MGMT is epigenetically silenced, this is irrelevant, since MGMT is not available for repair. The situation is different with the nitrosoureas, because p53 robustly upregulates the repair genes DDB2 and XPC [48]. The corresponding repair proteins are involved in nucleotide excision repair (NER) and crosslink repair by detecting major damage to the DNA and initiating the excision process [110]. This is irrelevant for TMZ-induced methylation damage, but is relevant for DNA chloroethylation and crosslinks induced by CCNU and other nitrosoureas (BCNU, ACNU, and fotemustine). The increased crosslink repair leads to drug resistance [48]. p53 also robustly upregulates translesion polymerase eta (POLH), which is involved in the repair and tolerance of DNA damage, including that induced by nitrosoureas [111]. Thus, we have shown that the upregulation of POLH is associated with the resistance of glioma cells to nitrosoureas [112]. POLH was also shown to be involved in the tolerance of O^6^MeG adducts and the formation of O^6^MeG/T mismatches [113]. Another translesion polymerase that we identified as conferring TMZ and nitrosourea resistance is Rev3L [114]. It would be challenging to determine the expression of these damage-tolerance polymerases in gliomas in relation to therapeutic success. 

(b) The phosphorylated form p53Ser46 regulates a number of target genes that are pro-apoptotic: FAS(CD95/APO1), BAX, BAK, PTEN, and PUMA [115]. Although there are cell-type-specific differences and genes are sometimes “silenced” in glioma cells, the studies show that p53 wild-type cells are more sensitive to methylating genotoxins than p53-mutated cells. These also undergo apoptosis after TMZ, but higher doses are necessary because the endogenous (mitochondrial) apoptosis pathway must be activated [47]. This way is less efficient and requires more DNA damage and DDR activation. Therefore, p53 appears to be pro-apoptotic (and pro-senescent) in glioma cells treated with TMZ. 

(c) p53 is found not only (as a tetrameric transcription factor) in the nucleus but also in the cytoplasm. Here, it can interact with proteins of the BCL-2 family and enhance mitochondrial apoptosis through translocation to the mitochondrial membrane and release of cytochrome C [116,117]. However, it is unclear whether this pathway is also activated in glioblastoma cells at therapeutic doses of TMZ and CCNU.

**Figure 4:**
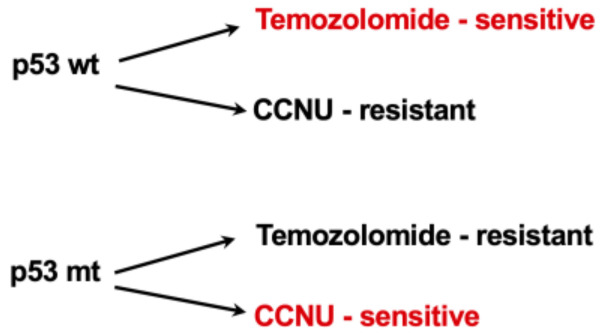
p53 as a determinant of TMZ sensitization and CCNU resistance. See text for explanation.

Taken together, experimental data indicate that p53 plays a dual role in activating survival and death functions. On the basis of these findings, we proposed that the therapy should be adjusted accordingly: gliomas with p53wt status should preferably be treated with TMZ, while those with p53mt (without transactivation activity of the protein) should be treated with CCNU or a combination of TMZ/CCNU [118] (Figure 4). Currently, the p53 status does not seem to be used routinely to make therapy decisions, and unfortunately, there are no corresponding clinical studies. The observed overall good response of patients to TMZ-CCNU-based therapy [16] could be due to the inclusion in the study of both p53wt and p53mt glioblastomas that are responding well to either one of the drugs.

## 16. Synthetic Lethality and Role of PARP

Synthetic lethality (STL; failure of one repair gene is tolerated by the cell, whereas failure of a second gene or gene product results in cell death) has been best studied in cells with mutant BRCA1 and BRCA2. These genes mutated in hereditary breast cancer are involved in DSB repair, the HR pathway. Downregulation of HR (BRCA2 or RAD51) resulted in glioblastoma cell sensitization to TMZ and ACNU, which was ameliorated in the presence of the PARP inhibitor olaparib [119]. Other approaches are encouraging. Thus, it has recently been shown that a brain-permeable PI3 kinase inhibitor together with HR or ATM inhibition causes synthetic lethality in glioma cells [120]. Although PARP-1 plays a role in detecting DNA strand breaks and regulating BER, NHEJ and chromatin remodelling [110], PARP-1 inhibitors are not toxic per se, but they strongly impact the sensitivity of mutant tumor cells defective in HR. Therefore, a glioma tumor screening for HR deficiency is warranted as a precondition of a trial of PARP inhibitor applications for glioblastoma monotherapy.

It is well known that PARP inhibition significantly increases the toxic effect of alkylating agents, which happens when BER comes into play, i.e., the toxic effects are brought about by N-alkylations. Therefore, coadministration of PARP inhibitors (olaparib, niraparib, rucaparib, and talazoparib) with TMZ or procarbazine is anticipated to ameliorate tumor cytotoxicity in the presence of MGMT. This is in accordance with recent findings. Thus, it has been shown that PARP inhibition enhances the toxic effect of TMZ in MGMT-proficient, but not in MGMT-deficient, cancer cells. At the same time the data shows that PARP-1 is not involved in the O^6^MeG response. Importantly, together with the downregulation of HR proteins such as BRCA2 and Rad51 [119] as well as XRCC3 [121], olaparib further increases the toxic effect of TMZ. In glioblastomas, in which these or other members of the HR gene family are downregulated, concomitant administration of TMZ with a PARP inhibitor is therefore expected to have synergistic effects. 

**Figure 5:**
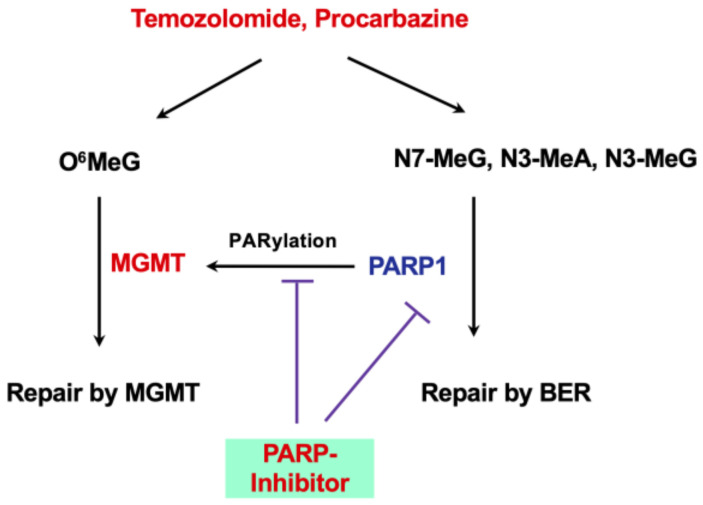
Effect of PARP inhibitors (olaparib and others) together with TMZ or procarbazine.

An interesting recent observation should not go unmentioned: PARP-1 interacts with MGMT; it binds to it and causes PARylation of the repair protein, thereby stimulating its activity, probably through increased chromatin binding. The inhibition of PARP-1 therefore likely leads to an accumulation of O^6^MeG, even in MGMT+ tumors, ameliorating the toxic effect of TMZ. This has been shown in vitro as well as in a mouse glioma model (PARP inhibitors are brain-permeable) [122,123]. Of note, TMZ together with PARP inhibition was without systemic toxicity and tolerable in normal tissues [122]. Therefore, cotreatment with PARP inhibitor and TMZ (or procarbazine) appears to be a reasonable strategy in the therapy of promoter-unmethylated gliomas with a bad prognosis (Figure 5).

## 17. Glucocorticoids and TMZ/Procarbazine Therapy

Glucocorticoids are being used in glioblastoma therapy to reduce tumor-associated cerebral edema, which is a common side effect of radiochemotherapy. Glioma patients who received standard therapy (RT+TMZ) concomitant with dexamethasone showed a worse outcome [124]. Interestingly, the reduction in OS in the TMZ group treated with dexamethasone could be abrogated by concomitant treatment with bevacizumab [124], indicating complex pathways involved. Although MGMT promoter methylation was not stated in this study, it is reasonable to suppose that glucocorticoids cause upregulation of MGMT as the *MGMT* gene harbors two GREs in the promoter [125]. Actually, a comparative study of MGMT promoter activity in GBM cell lines revealed *MGMT* induction following dexamethasone, but not upon TMZ and radiation treatment. The epigenetic status was not altered following TMZ and RT [77]. It should be noted that corticosteroids shorten patient survival following RT-TMZ and also in glioma-bearing mice following RT only [78], indicating mechanisms involved that target not only O6AA pathways. Overall, the data strongly suggest a restricted use of corticosteroids in alkylating agent/RT-based GBM therapy.

## 18. Interaction of Radiation with TMZ

Postoperatively, radiation therapy (RT) combined with TMZ (according to the Stupp scheme) is the gold standard in the treatment of glioblastoma [5,126]. Usually, 60 Gy are administered in two Gy fractions and 75 mg/m^2^ TMZ. In this adjuvant setting, TMZ is considered a radiation sensitizer. However, both RT and TMZ induce DSB and are potent inducers of apoptosis, cellular senescence, and autophagy, suggesting that radiation and TMZ act independently. Interestingly, there are no study-based binding guidelines for the timing of treatments. Thus, it is unclear whether TMZ should be given before or after radiation. As a rule, TMZ is taken before radiation, but this is more for “technical” reasons (the drug is taken at home in the morning before the radiation takes place in the clinic). Since RT and TMZ are genotoxic exposures, the cooperative effect of RT and TMZ can only be understood by unrevealing the molecular processes in tumor cells at the DNA level. Cell culture experiments indicate that the cytotoxic effect of RT is only enhanced when TMZ is administered prior to irradiation. A synergistic effect of RT and TMZ was independent of MGMT status [127], suggesting that lesions other than O^6^MeG are involved. 

To explain the interaction between RT and TMZ, it should be considered that DNA replication is essential for TMZ, which is blocked by radiation. It is therefore possible that the proliferation index decreases after repeated RT, and the TMZ effect is thus attenuated. A synergistic effect of RT and TMZ can be explained by the interaction between methylation and radiation damage. The main DNA adducts produced by TMZ are N7-methylguanine, N3-methylguanine, and N3-methyladenine, that of radiation 8-oxo-guanine. All these adducts are removed by BER, where high adduct levels lead to DSBs in overlapping repair patches. This would explain the sensitizing effect of radiation exposure, which is independent of MGMT. These and other scenarios have been previously discussed [128]. Based on the data on the interaction of alkylation and oxidative lesions, it seems reasonable to take TMZ 2–4 h before RT (maximum serum levels are reached 3–4 h after TMZ intake; for references see [71]) to optimize the effects. It should be noted that radiation causes replication inhibition that counteracts S-phase-dependent O6AA. Therefore, it is worth considering the option to precede RT/TMZ therapy with a TMZ monotherapy boost.

## 19. TMZ and TTF Therapy

Therapy with electric fields (tumor-treating electric fields, TTF, and 100–200 kHz) has attracted considerable attention in recent years. In vitro, TTF potentiates the cytotoxic effect of TMZ in (patient-derived) glioblastoma cells [129], and TTF treatment together with adjuvant TMZ increased both the progression-free interval and overall survival [130]. The synergistic effect with TMZ proved to be independent of the MGMT status, which was shown in several studies in vitro. Disruption of the mitotic spindle and thus mitotic cell death [131] as well as inhibition of repair processes, in particular DSB repair by HR, have been described mechanistically [132,133]. This has been verified in a recent study on GBM cell lines, demonstrating enhanced toxicity following TMZ or CCNU treatment in the presence of TTF [134]. Since the HR pathway, which is key in the resistance to TMZ and crosslinking nitrosoureas, is only active in proliferating cells, increased toxicity is expected to be limited to the proliferating tumor population, without site effects to the normal (non-proliferating) tissue. TTF seems therefore to be a useful support, not only in TMZ-based therapy, but for alkylating agents in general, both in the primary and in recurrent situations. In a phase 3 trial, Lazaridis et al. showed that CCNU plus TMZ together with TTF for at least eight weeks was associated with prolonged survival, compared to CCNU/TMZ only [135]. These encouraging results need further confirmation by clinical studies.

## 20. CUSP9v3 Therapy 

In this therapy setting, TMZ is administered in low doses (20 mg/m^2^) twice a day. Additionally, there are a number of pharmaceuticals that are intended for the therapy of other diseases (repurposed drugs) and attack very different cellular targets [136]. As confirmed by a phase 1 study, CUSP9v3 therapy appears to be well tolerated [137]. The low dose of TMZ and twice-daily administration protocol is reasonable, since damage accumulation is to be expected in MGMT-deficient tumors (see explanations above) and side effects are kept to a minimum. A mechanistic explanation for a possible cooperative effect of the pharmaceuticals with TMZ-induced signaling pathways requires further investigation.

## 21. Immunotherapy (Together with O6AA)

TMZ is a powerful mutagen. In the surviving proliferating population, O^6^MeG constitutes a source of gene mutations that ensure tumor evolution (Figure 2). It is easy to imagine that this will create new tumor epitopes that could be of interest for immunotherapeutic approaches. Numerous studies evaluated the combinatory effect of checkpoint inhibitors (CPIs) together with alkylating chemotherapy for the treatment of glioblastoma [138]. Today, adjuvant addition of CPIs failed to improve survival in glioma patients [139]. This might be partially explained by the finding that hypermutation after TMZ is phenotypically distinct and causes immune rejection independent of the PD-1/PD-L1 axis [140], supporting the view that the brain microenvironment is immunosuppressive. However, given that neoadjuvant CPIs are prior to resection (unmasking even more potential tumor epitopes and reducing tumor burden), alkylating chemotherapy might present a window for a beneficial use of CPI in combination with TMZ or CCNU [140]. In addition to this, drug-induced xenogenization with an RT-triggered abscopal effect was proposed as a potential therapeutic modality [141].

## 22. CDK Inhibitors

Cyclin-dependent kinases (CDKs) are key nodes in cell cycle regulation; they are involved in many processes driving tumor progression such as autonomous tumor growth, angiogenesis, growth factor receptor signaling, and tumor immunity. A couple of clinical phase I and II trials have been conducted with CDK inhibitors alone or in combination with GBM standard therapy [142]. The most encouraging was a clinical trial (REGOMA) that identified the multi-kinase inhibitor regorafenib (Strivaga) as efficient and safe in the treatment of recurrent GBM, showing a better patient survival than CCNU only [143]. These results, as promising as they are, did not remain unchallenged as the REGOMA study referred to a historical cohort of patients treated extensively with O6AA including CCNU. Still, confirmation was found in a recent trial, revealing at the same time that dose reduction was effective in attenuating toxic side effects without reducing the therapeutic benefit [144]. In these studies, the CDK inhibitor was administered after chemotherapy. It should be noted that coadministration with TMZ or PCV is expected to be counterproductive as the alkylating agents require tumor cell proliferation to be effective, at least in the therapeutic dose range. Glioblastoma with alterations in the MAPK pathway respond poorly to regorafenib treatment, whereas EGFR-altered tumors with expression of EGFRvIII show a better response [109]. In vitro experiments revealed that regorafenib limits cell growth, but is ineffective in inducing apoptosis [145].

## 23. Why Are Glioblastomas Refractory to Alkylating Agent-Based Therapy?

There are certainly several reasons why glioblastomas are difficult to cure, such as the inability to completely remove the tumor surgically, the tumor’s infiltrative growth, its immunological properties, and the (supposed) inherent radiation and chemoresistance. Key mechanisms contributing to TMZ/procarbazine/CCNU resistance are briefly summarized below. 

(a) Proliferation/cell division. The effect of TMZ, procarbazine, and most likely also of CCNU and other alkylating agents in the therapeutic dose range is S-phase-dependent and thus bound on cell proliferation [146]. The proliferating fraction in glioblastomas has been determined to be about 22% (Ki-67 positive cells) [147]. Dormant tumor cells are not affected by O6AA. A positive correlation between Ki-67 labeling index and overall survival has been demonstrated [147,148]. However, there are also contrary data, which are possibly due to the fact that promoter-methylated and unmethylated cases were not recorded separately in these studies. It is important to note that all genotoxic treatments cause a transient block of replication, which may impact the response in long-term treatment schedules.

(b) Repair through MGMT. Under therapeutically relevant conditions, intracerebral TMZ levels of 1 to 10 µM can be achieved inducing less than 10,000 adducts per cell [71]. Thus, small amounts of MGMT (<10,000 molecules per cell) may already cause protection. There seems to be a threshold: patients with low MGMT tumor levels (≤30 fmol/mg protein) responded better to TMZ than patients with MGMT activities above this level. This correlation was not seen in patients who only received RT [24]. It is important to note that in MGMT-deficient glioblastoma cells the effects of low doses accumulate with repetitive treatments [149]. 

(c) Repair through BER. TMZ-induced N-alkylations (N7-methylguanine, N3-methyladenine, and N3-methylguanine) are toxic, as they inhibit replication directly or indirectly through apurinic sites during their repair [150]. N3-methyladenine, N3-methylguanine and N7-methylguanine are removed by the N-methylpurine-DNA glycosylase (MPG), leaving an AP site, which is subsequently repaired. It was shown that BER intermediates strongly block replication by producing DSBs and chromosomal changes [151]. Therefore, BER intermediates seem to be the trigger of genotoxicity. This is in line with a report demonstrating that MPG overexpression together with the inhibition of downstream steps of repair sensitize glioma cells to TMZ [152]. MPG is often overexpressed in glioblastomas, and a correlation was shown between high MPG expression, TMZ resistance, and poor OS compared to patients lacking MPG (as measured by immunohistochemistry) [153]. MPG is found in immunostainings in the cytoplasm and nucleus. High nuclear MPG levels (associated with epigenetic silencing of HTATIP2, a negative regulator of cytoplasmic-nuclear translocation) appear to be associated with resistance to methylating agents [154]. Drug screening revealed Sunitinib to be effective in inhibiting MPG, which restored TMZ sensitivity [155], suggesting repurposing the drug in glioma therapy. Overall, in MGMT proficient-tumors, imbalances in BER may contribute to drug resistance as well as sensitivity. In MGMT-lacking tumors, the contribution of N-methylations to toxicity appears to be low compared to O^6^MeG, because the adduct is stable and remains in the DNA, causing a constant source of MMR-mediated DSBs. Importantly, there is a crosstalk between MGMT and BER, since PARP1 is activated during BER and this, in turn, increases the effect of MGMT through PARylation (Figure 5). Consequently, inhibition of BER would also affect MGMT-mediated repair, which was shown experimentally [123]. 

(d) Mismatch Repair. MMR is essential for the cytotoxicity and genotoxicity of TMZ, procarbazine, and other O^6^-methylating anticancer drugs (see Figure 3). The proteins MSH2, MSH6, MLH1, PMS2, and EXO1 are involved in MMR. Down-regulation of MLH1 and PMS2 [156] as well as MSH6 [157] has been described in gliomas, which does not lead to complete loss, but rather to quantitative variability in expression. This correlates with drug sensitivity [157]. Clinical studies, on the other hand, convey a heterogeneous picture. Thus, it was reported that MMR deficiency does not lead to clinical resistance to TMZ in gliomas [158], while a study published later showed that even small changes in the expression of MSH2 have an influence on TMZ sensitivity and thus, the therapeutic success [159]. The latter study is compatible with in vitro data showing a gene dose effect [157]. It should be stressed that an influence of MMR will only be visible in MGMT-deficient tumors treated with methylating drugs and not for CCNU-based therapy. A comprehensive analysis of mutational signatures of a large number of gliomas revealed that TMZ-sensitive tumors frequently acquire TMZ resistance due to MMR defects, which is associated with poor patient survival (and low response to immunotherapy) [160]. Overall, MMR is strongly deregulated in gliomas having a significant impact on therapy with O6-methylating drugs. 

(e) Double-strand break (DSB) repair. The ultimate toxic lesions after radiation and alkylating agent exposure are DSBs. Their repair is consequently an important resistance factor. DSBs are repaired by NHEJ and homologous recombination (HR). HR is a complex process that also plays a role in overcoming DNA replication block, tolerance of DNA lesions, and crosslink repair. Cell culture model systems showed that NHEJ mutants (DNA-PK) are hardly sensitive to TMZ, while mutations in one of the HR genes led to a drastic sensitization of the cells [161]. Apparently, HR is crucially involved in the tolerance of O^6^ MeG-generated secondary lesions. We have shown experimentally that downregulation of HR genes (BRCA2, RAD51) leads to TMZ sensitization [119], and pharmacological inhibition of RAD51 causes sensitization to CCNU, which was verified in a mouse xenograft model [161]. Clinical trials on glioblastomas with HR inhibitors are pending. 

(f) DNA damage response. The ATR/ATM-CHK1/CHK2/HIPK2-p53 signaling pathway is activated in glioblastoma cells after treatment with TMZ [45]. It is interesting that the inhibitors of HIPK2 and SIAH1 are often overexpressed in glioblastoma [45], which would impede activation of HIPK2 after DNA damage. Therefore, inhibitors targeting this pathway are anticipated to be useful to achieve better activation of the apoptotic pathway. ATM and ATR mutant cells are hypersensitive to TMZ, and pharmacological inhibition of ATM or ATR after TMZ treatment of glioblastoma cells resulted in an increase in the rate of apoptosis [43], which led to an indication that repair proteins (including BRCA2) are activated via the damage response triggered by TMZ. The inhibition of ATR (and CHK1) proved to be more efficient than ATM (CHK2), which is presumably due to the dual role of ATM, which includes proapoptotic HIPK2 activation. Recently, RAD18 was shown to be involved in the DDR, having an impact on the TMZ sensitivity of glioma cells and patient response [162]. The pro- and antiapoptotic roles of p53 have been described above. Cells with deficient or functionally inactivated p53 also undergo apoptosis, but higher doses of TMZ are required. In this case, apoptosis occurs through activation of the mitochondrial pathway, which is less efficient in the absence of p53 [47]. In view of the heterogeneity of glioblastomas with regard to MGMT and p53 status, the simultaneous administration of TMZ or procarbazine and CCNU according to the PCV protocol (procarbazine, CCNU, and vincristine) or concomitant treatment with TMZ and CCNU, according to Herrlinger et al. [163], is a reasonable strategy.

(g) Autophagy and apoptosis regulators. TMZ triggers low-dose autophagy that counteracts apoptosis, and abrogation of the autophagy pathway ameliorates the killing effects of TMZ [49]. This indicates the need for autophagy inhibition strategies. The induction of apoptosis is a complex process in which proteases (caspases) and modulating proteins play a critical role. TMZ activates the downstream caspases-3 and -9, which cleave the inhibitor (iCAD) of the caspase-activated DNAse DFF40/CAD and thus initiate DNA fragmentation—the most important hallmark of apoptosis [164]. The inefficiency of glioblastoma cells to undergo apoptosis is often considered to be the cause of intrinsic resistance, and it is obvious that it is causally linked to downregulation or the lack of critical apoptosis factors. In fact, it has been shown that in glioblastomas and grade 4 astrocytoma in situ and cell lines derived from them, DFF40/CAD is hardly expressed, which means that DNA fragmentation does not take place, despite activated caspase-3 and -9. Therefore, apoptosis cannot be completed [165]. The low expression of DFF40/CAD, also shown on tumor specimens [165], provides a reasonable explanation for the intrinsic radio- and chemo-resistance of glioblastoma. 

(h) Therapy-induced cellular senescence (TIS). Given the inefficiency of glioblastoma cells to die from apoptosis, it is reasonable to suppose that kinases activated by DNA damage activate alternative pathways. One of these leads to senescence (Figure 3). Indeed, in glioblastoma cells, senescence is the quantitatively predominant event caused by TMZ-induced DNA damage [55], and the upstream signaling pathways elicited by the critical damage O^6^MeG are identical for apoptosis and senescence, bound on active MMR [23]. Extrapolated to the in vivo situation, it is reasonable to suppose that the high rate of senescence in glioblastoma prevents tumor cells from being eliminated. Clinically, this would result in a stable disease, provided the senescent compartment of the tumor remains in the dormant stage.

## 24. Can Senescent Cells Become Reactivated to Proliferate? 

By definition, senescent cells are irreversibly blocked in the cell cycle. However, there is increasing evidence that there are exceptions to the rule and that therapy-induced senescent cells (TIS) can be reactivated to proliferate. Therefore, some authors designate these senescent cells as “pseudo-senescent”. If these non-dividing (and therefore TMZ-resistant) cells became reactivated, we would be dealing with a recurrence. A reactivation of tumor cells from a senescent stage (induced by etoposide) was experimentally demonstrated. It was associated with a weakening of the SASP as well as an increase in the polyploidy state and aggressive tumor growth [166]. Reactivation from quiescence, in which cells can remain for long periods (months, years), appears to be a rare event; in a lung carcinoma model, it was determined to be 1 in 10^6^ cells [167], which corresponds to a gene mutation rate. A release from the quiescent stage, months or years after cytotoxic therapy (as well as cell cycle blockers), would inevitably result in late tumor recurrence. In principle, a single reactivated cell is sufficient to clonally form a tumor again. In summary, reactivation of senescent cells represents a plausible explanation for recurrence. 

Although data are still lacking, it is very likely that this scenario also applies to glioblastoma. It is reasonable to suppose that the induction of senescence takes place already after the first RT/TMZ treatments, leading to a highly resistant tumor subpopulation that is still present in the recurrence [55]. It is very likely that this exhibits the SASP that was shown to be associated with TMZ-induced senescence in vitro [56]. As therapy-induced senescent cells secrete pro-inflammatory cytokines and modulate the immune system, thereby evading immune surveillance [57,58], they worsen the clinical outcome. Overall, there is accumulating evidence that therapy-induced senescence plays a role in glioma recurrence and progression [168].

## 25. Do We Need Senolytics and Senostatics? 

Senescent cells do not divide and display an upregulation of antiapoptotic functions. They are, therefore, refractory to therapy, notably with S-phase-dependent drugs. They drive tumor progression through SASP and represent a potential source of recurrent tumor growth. Therefore, treatments with senotherapeutics that suppress SASP (senomorphics) or selectively kill senescent cells (senolytics) are highly desirable. We tested a number of compounds and natural products for their senolytic potential in glioblastoma cells and were able to show that known senolytics such as ABT-737 and ABT-263 (Navitoclax) kill TMZ-induced senescent cells but not proliferating glioblastoma cells. We also found that chloroquine, the inhibitors of ATM and ATR, BV-6, and PX-866 are senolytic [50]. Targeting of the senescence-associated antiapoptotic pathway involving c-IAP2 and Bcl-2 by a combination of BV-6 and venetoclax synergistically ameliorated senolysis [169].

Of the natural substances investigated, we found fisetin and artesunate to have senolytic activity on glioblastoma cells [50]. This finding is particularly interesting because both natural substances are well known as dietary supplements and turned out to be tolerable even after long-term uptake. Artesunate is a derivative of artemisinin—the pharmacologically active agent from *Artemisia annua* [170]. We have shown that artesunate produces a sustained level of intracellular ROS, leading to the formation of oxidative DNA damage, DSB, and ultimately cell death [171,172]. The presence of iron promotes this autocatalytic process [173]. Artesunate also inhibits repair processes (homologous recombination) and senescence induction and thus enhances the effect of TMZ [174]. The exact mechanism by which it acts as a senolytic (presumably through ROS) is not known. 

Fisetin is a flavonoid and polyphenol found in smoke bush (Fiset wood), various fruits (apples, strawberries, grapes, persimmons) and vegetables (cucumbers, onions). Like artesunate, it is genotoxic in high doses in glioblastoma cells (unpublished data). Fisetin proved to be an effective senolytic in various tumor cell systems [175], and the oral administration of fisetin led to a prolongation of the life span in mice, most likely due to the selective killing of aged cells [176]. Fisetin has, therefore, gained popularity as a food supplement and senolytic agent consumed by healthy people. The senolytic effect of artesunate and fisetin on glioblastoma cells suggests that these natural senotherapeutic agents might be recommended to patients after standard RT/TMZ and during adjuvant TMZ as supportive therapy. [177]. CCNU and RT themselves do not have senolytic activity [78]. Therefore, they cannot be used to eliminate TMZ-induced senescent cells. It should be noted that the most effective senolytic drug combination to date is dasatinib (Sprycel) and quercetin [177].

## 26. Conclusions and Summary

The signaling pathways triggered by the critical lesion O^6^MeG induced by TMZ, procarbazine, and other methylating agents have been well studied. The amount of O^6^MeG increases linearly with dose in MGMT-lacking glioblastoma cells, and the DNA damage response is activated to the same extent. This also applies to the end-points apoptosis and senescence in glioblastoma cells, so it can be concluded that even low doses are effective. In addition, in the absence of MGMT, there is an accumulation of critical damage upon repetitive TMZ administration, leading to an enhanced cellular response. These experimental findings support metronomic therapy protocols, at least for promoter-methylated cases. 

Studies show that TMZ is suitable for long-term therapy (up to 101 cycles have been reported) in newly diagnosed glioblastoma [178]. The low efficiency of RT/TMZ and adjuvant TMZ may nevertheless be due to the fact that (a) not all tumor cells proliferate with the resting fraction to be therapy-refractory, (b) MGMT is expressed but below the detection level, (c) MMR is down- and HR up-regulated, (d) glioblastoma cells inefficiently complete apoptosis, and (e) the majority of proliferating tumor fraction goes into therapy-induced senescence. This is associated with SASP, which is pro-inflammatory and promotes tumor progression. 

There is increasing evidence that senescence is not an absolute dormant stage, but (with low frequency) senescent cells can become reactivated to proliferate. It is reasonable to conclude that this is a key mechanism resulting in the formation of recurrences. Consequently, supportive administration of senotherapeutics during and after TMZ or CCNU/procarbazine therapy is desirable. Fisetin and artesunate are among the natural substances that were shown to bear senolytic activity on glioblastoma cells [50]. They are well tolerated and are taken as food supplements by a wide range of people. It is possible that these natural substances are effective in adjuvant therapy together with quercetin and curcumin, administered supportively in intervals. Clinical trials with the potent senolytic combination dasatinib and quercetin administered (hit-and-run) during and after O6AA therapy are warranted.

Since the critical toxic and senescence-triggering primary lesions induced by TMZ, procarbazine, and CCNU are repaired by MGMT, the repair protein deserves special attention in terms of inhibition strategies. MGMT inhibitors (O^6^-BG and lomeguatrib alias PaTrin-2) are highly efficient and free of side effects. Tumor targeting could be achieved by intracranial application, which would reduce systemic side effects, as only the proliferating tumor fraction is targeted by concomitant O6AA therapy, and hematotoxicity is expected to be negligible. Inhibitors of PARP-1 are known to mediate synthetic lethality. Although hereditary DNA repair defects (involving the BRCA genes) have not been described to play a role in gliomas, downregulation or pharmacologic inhibition of HR or PARP-1 may be a reasonable strategy, notably for MGMT-expressing tumors. Of note, PARP-1 interacts with MGMT and thus increases its effect, which is attenuated by PARP inhibition. Furthermore, p53 deserves attention because it upregulates repair genes known to cause CCNU resistance and proapoptotic genes that enhance the anticancer effect of methylating drugs.

The PCV therapy regimen is often used for grade 3 tumors. Since procarbazine acts like TMZ at the molecular level, experimental data obtained with TMZ can be extrapolated to procarbazine. Procarbazine becomes activated by Cyp3A4, and polymorphisms and drug interactions should be taken into account. The co-administration of CCNU has the advantage of inducing cell death in MGMT-deficient tumors through an MMR-independent pathway, increasing the likelihood of an effective strategy. Co-administration of vincristine captures tumor cells that have escaped proliferative arrest and cell death following DNA alkylation. It is important to note that TMZ and PCV therapy only affect proliferating cells. Therefore, strategies that target the slowly proliferating tumor cell population and/or activate tumor cells to proliferate (we have shown this experimentally using the mitogen PMT [47]) are desirable. 

Like CCNU, BCNU (carmustine) has chloroethylating capacity, but as a bifunctional alkylating agent, it also exerts a direct crosslinking effect. BCNU is sometimes used as a Gliadel wafer mounted on a substrate for intracerebral therapy after primary surgery. The protective effect of MGMT on BCNU is lower compared to CCNU (or ACNU), and because of the direct crosslinking activity, stronger side effects on the healthy tissue are expected for BCNU. CCNU and/or TMZ or derivatives immobilized on a carrier useful for intracerebral administration, would represent an alternative choice.

Current ongoing studies will reveal whether TTF-based therapy together with TMZ in the recurrence situation is effective. The mechanism of TTF known so far indicates that it attacks proliferating cells. This is even more true when TTF is used together with TMZ for therapy. Whether slowly proliferating tumor cells or dormant (senescent) cells can be eliminated by TTF is an open question. Natural substances such as artesunate, which produce ROS intracellularly, thus causing a sustained level of oxidative DNA damage in tumor cells [171], may be suitable together with TTF for eliminating this tumor subpopulation.
